# Nonlinear dimension reduction with Wright–Fisher kernel for genotype aggregation and association mapping

**DOI:** 10.1093/bioinformatics/bts406

**Published:** 2012-09-03

**Authors:** Hongjie Zhu, Lexin Li, Hua Zhou

**Affiliations:** ^1^Department of Psychiatry and Behavior Science, Duke University, Durham, NC 27710; ^2^Department of Statistics, North Carolina State University, Raleigh, NC 27695, USA

## Abstract

**Motivation:** Association tests based on next-generation sequencing data are often under-powered due to the presence of rare variants and large amount of neutral or protective variants. A successful strategy is to aggregate genetic information within meaningful single-nucleotide polymorphism (SNP) sets, e.g. genes or pathways, and test association on SNP sets. Many existing methods for group-wise tests require specific assumptions about the direction of individual SNP effects and/or perform poorly in the presence of interactions.

**Results:** We propose a joint association test strategy based on two key components: a nonlinear supervised dimension reduction approach for effective SNP information aggregation and a novel kernel specially designed for qualitative genotype data. The new test demonstrates superior performance in identifying causal genes over existing methods across a large variety of disease models simulated from sequence data of real genes. In general, the proposed method provides an association test strategy that can (i) detect both rare and common causal variants, (ii) deal with both additive and interaction effect, (iii) handle both quantitative traits and disease dichotomies and (iv) incorporate non-genetic covariates. In addition, the new kernel can potentially boost the power of the entire family of kernel-based methods for genetic data analysis.

**Availability:** The method is implemented in MATLAB. Source code is available upon request.

**Contact:** hongjie.zhu@duke.edu

## 1 INTRODUCTION

Genome-wide association studies (GWASs) based on single-nucleotide polymorphism (SNP) chips have enjoyed varying degrees of success in identifying genes associated with complex diseases or traits (Easton and Eeles, 2008; Frazer *et al.*, 2009; Lettre and Rioux, 2008). It has now been widely accepted that standard GWAS explains at most a small fraction of the population variation of most complex traits (Frazer *et al.*, 2009). Recently, deep resequencing is emerging as a new and potent means for mapping complex trait genes. Resequencing delivers orders of magnitude more variants than SNP chips and include both common variants with minor allele frequency (MAF) *>*10%, as well as rare variants with MAF *<*1%. Availability of rare variant information presents unique opportunities to evaluate the ‘common disease rare variants’ hypothesis. This hypothesis states that a complex disease can be attributed to multiple rare variants with relatively high risks, and it has attracted much attention in recent studies. Attesting to this hypothesis, a number of deleterious or protective rare variants have been identified for low plasma levels of high-density lipoprotein cholesterol (Cohen *et al.*, 2004), hypertension (Ji *et al.*, 2008) and Type-I diabetes (Nejentsev *et al.*, 2009).

Identifying disease associated rare variants, however, is challenging, because a particular rare disease predisposing allele may be present in only a handful of patients. Henceforth, traditional single marker tests that capture only marginal effects are doomed to have low power. A useful strategy to address this challenge is to effectively merge information in SNP variants by some meaningful SNP sets, for instance, genes or pathways, and then to identify disease-associated genes or pathways rather than disease variants. Following this idea, several aggregation-based association test approaches have been developed. Li and Leal (2008) proposed a group-wise test exploiting both multivariate and collapsing strategies that possess higher power than a simple multivariate test or simple collapsing. Madsen and Browning (2009) extended the method by incorporating weights that depend on MAF into the group-wise statistics and approximating *P*-values by permutations within each group. Both methods consider rare variants with MAF falling below a pre-specified threshold and exclude common variants from analysis. This separate treatment seems counterproductive because in reality both common and rare variants can be informative. The pooling strategy of Price *et al.* (2010) circumvents the issue of arbitrarily chosen frequency threshold by calculating a group-wise statistic under a variety of thresholds. However, this strategy also has several limitations. First, environmental predictors are excluded from analysis even though they may contribute significantly to a disease. Second, interactions among SNPs cannot be effectively detected. Third, the solution is sensitive to the classification of variants: if all types of variants, deleterious, protective or neutral, coexist, then various signals can cancel one another during the pooling and thus can potentially compromise statistical power. Liu and Leal (2010) proposed a SNP set genotype-based statistic for rare variants and declared that the common variants and environment factors can be modeled together with the rare variant statistic in a logistic regression (LR) model. However, interactions between rare and common variants still cannot be explicitly modeled in this way, and the method handles only dichotomous traits.

The field of statistical dimension reduction (DR) offers a useful and appealing means for genotype aggregation. It is based on the belief that high-dimensional data can be effectively summarized in a low-dimensional space, and the subsequent modeling can concentrate on the reduced space. The most commonly used dimension reduction approach is principal components analysis (PCA). Chen *et al.* (2010) applied PCA to combine SNP information within pathways, generated the so-called eigen-SNPs, and used eigen-SNPs in subsequent association mapping. However, PCA has at least two limitations. First, PCA aggregates SNPs regardless of the phenotypic trait information. Since mapping traits to associated genes is of ultimate interest, it is intuitively desirable to aggregate SNPs under the guidance of trait information such as disease status or quantitative traits. In statistical terms, PCA is an ‘unsupervised’ DR solution, whereas a ‘supervised’ DR solution is preferred. Second, the eigen-SNPs, or principle components, are ‘linear’ combinations of the SNPs. As a consequence, such summary measures may fail to capture complex interacting effects among the individual SNPs, and in turn reduce the power of subsequent association mapping.

In this article, we develop a powerful association mapping approach based on next-generation sequencing data. Two key ingredients of the proposed method are a statistical DR that achieves supervised and nonlinear reduction, and a new kernel that is based on Markov chain theory and particularly suitable for qualitative SNP data. Our contributions are mainly 2-folds. First, the proposed association mapping approach simultaneously takes into account (i) both rare and common variants, (ii) both additive and interaction effect, (iii) quantitative traits as well as disease dichotomies and (iv) non-genetic covariates. Second, the commonly used kernels, such as Gaussian, polynomial and spline, work successfully with continuous attributes, but may perform poorly for discrete genetic data. The proposed new kernel is specially designed for discrete attributes and can effectively capture the similarity between individual genotypes. Moreover, the new kernel is novelly derived from powerful Markov chain theory and can benefit many kernel-based learning methods in general. We compare our proposal with some state-of-the-art aggregation and mapping solutions and find that our method clearly achieves superior power in a variety of different genetic model scenarios.

The remainder of the article is organized as follows. Section 2 describes our proposed association mapping, including kernel-based nonlinear DR and construction of new kernels. Section 3 presents numerical studies comparing various aggregation and association mapping solutions. Section 4 concludes the article with a discussion and suggests potential future extensions.

## 2 METHODS

Suppose *n* study individuals are genotyped at a SNP set (e.g. a gene) that is composed of *p* SNPs denoted by *X*_1_,...,*X_p_*, and the trait *Y* can be either binary (case–control study) or quantitative. Potential non-genetic covariates, such as sex, age smoke, and are denoted by *C*_1_,...,*C_t_*. There can be multiple SNP sets, and we treat ‘one set at a time’. The goal is to test the association of the trait and all markers in the SNP-set ‘jointly’, after adjusting for non-genetic covariates. In sequence studies, the number of markers *p* is potentially large and can outnumber the number of subjects *n*. Our proposed group-wise association test consists of two key elements: a nonlinear supervised DR method that aggregates markers and produces summary features and a novel kernel that encodes genomic similarity. The nonlinear DR method was first proposed in Zhu and Li (2011) for gene pathway analysis and for completeness, we review the method here. The new kernel is constructed under the guidance of Markov chain theory for discrete genotype. Using Markov chains to build kernels for non-standard data is novel and the resulting kernels can potentially benefit the entire family of kernel methods (e.g. support vector machines) for genetic data analysis.

### 2.1 Nonlinear supervised DR

We begin with a brief review of PCA, which has been an extremely popular tool in analysis of genetic and genomic data. For instance, PCA has been used to adjust for population stratification (Price *et al.*, 2006) in GWAS or to produce a set of eigen-SNPs for association mapping (Chen *et al.*, 2010). Given *p* SNPs, PCA seeks linear combinations of SNPs that have maximal variances. It is solved by an eigen decomposition of SNP covariance matrix. Then, the eigenvectors with leading eigenvalues give the coefficients of linear combinations being sought. The linear transformed SNPs form the eigen-SNPs used in the subsequent analysis. Despite its widespread applications, however, PCA conducts DR without utilization of the phenotypic trait information, and thus there is no guarantee that the top extracted principle components are relevant to the traits. Consider a simple illustrative example. Suppose two SNPs *X*_1_ and *X*_2_ are in linkage disequilibrium (LD). Then, the variance of the linear combination *X*_1_ + *X*_2_ is larger than that of *X*_1_ − *X*_2_ and thus the eigen-gene found by PCA will be closer to the direction *X*_1_ + *X*_2_ than to *X*_1_ − *X*_2_. If in truth these two SNPs have opposite effects—one deleterious the other protective—then the trait depends on the SNPs through *X*_1_ − *X*_2_ and the eigen-gene would contain no signal for association.

Intuitively, it is natural to incorporate the trait information during the phase of DR, and this leads us to the family of ‘supervised’ sufficient dimensional reduction (SDR) approaches. For a regression of a response *Y* given a *p*-dimensional predictor *X*, SDR seeks a minimum number of linear combinations, *η*_1_^T^*X*,...,*η_d_*^T^*X*, such that
(1)
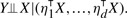

That is, *Y* depends on *X* only through those linear combinations, and one can replace the original *p*-dimensional *X* by now *d*-dimensional *η*^T^*X*. In practice, *d* is often much smaller than *p*, and thus DR is achieved. We call (*η*_1_^T^*X,*..., *η_d_*^T^*X*) the ‘linear sufficient predictors’, which will serve as the induced summary features in subsequent analysis. There have been many methods proposed for SDR, many of which can be formulated as a generalized eigen decomposition problem. Specifically, a reduction estimate can be obtained by the first *d* eigenvectors *η_j_*s that correspond to the nonzero eigenvalues *λ_j_*s in a descending order from the decomposition: *Ω_x_η_j_* = *λ_j_Σ_x_η_j_, j* = 1,...,*d*, where *Σ_x_* = Cov(*X*) and *Ω_x_* is a method-specific *p*×*p* semi-positive definite matrix (Zhu and Li, 2011). For instance, sliced inverse regression (SIR) (Li, 1991) is a widely used SDR estimator, where *Ω_x_* = Cov{E(*X*|*Y*)−E(*X*)}. This family of DR methods differ from PCA in that the response information is used in the DR phase. It is also interesting to note that all those SDR methods impose no parametric assumption on *Y*|*X*. Instead, they require the marginal distribution of *X* to be elliptically symmetric. This is often viewed as a mild condition, since it holds approximately when *p* goes to infinity. We assume the condition holds since we are dealing with a very large *p*.

The above SDR methods yield ‘linear’ DR, because the reduction admits the form of linear combinations of *X*. This could have some limitations. Consider an illustrative example, where *X* = (*X*_1_,...,*X*_6_) and *Y* = *X*_1_ + *X*_2_*X*_3_ + *X*_4_^2^ + *X*_5_*X*_6_ + *ε*, with an independent error *ε*. In this case, no linear reduction is possible. Another potential limitation is that one needs to invert a *p*×*p* covariance matrix *Σ_x_*, whereas its sample estimator is not invertible when *p* exceeds the sample size *n*. These observations motivate us to consider a ‘nonlinear’ DR strategy.

The basic idea is to use a function *ϕ*(·), with an associated kernel matrix *K*, to map *X* to *ϕ*(*X*). One then performs a linear DR in the space of *ϕ*(*X*), which in effect results in a nonlinear DR in the original predictor space 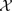. The well-known kernel trick turns the primal problem that depends on the dimension of the space of *ϕ*(*X*), which is high or even infinite, to a dual problem that only depends on the sample size. Consequently, the method works for *n < p*. Specifically, in analogy to linear reduction in Equation (1), nonlinear DR seeks
(2)
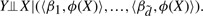

Comparing with Equation (1), the linear combinations (*η*_1_^T^*X,...,η_d_*^T^*X*) are replaced by the inner products 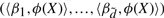 , and *Y* depends on *X* only through those inner products. We refer them as the ‘nonlinear sufficient predictors’ and assume the number 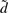 of inner products ≤ min(*n,p*). In terms of estimation, conceptually, one can estimate *β_j_*s in a way analogous to linear reduction, i.e. through the eigen decomposition: 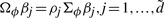 , where Σ*_ϕ_=* Cov{*ϕ*(*X*)} and Ω_ϕ_ is defined similarly as Ω*_x_* except we replace *X* with ϕ(*X*). Given {(*x*_1_,*y*_1_),...,(*x_*n*_*,*y_n_*)}, estimation of *β_j_*s is obtained by substituting in the corresponding sample counterparts: 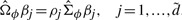 . On the other hand, the dimension of the induced mapping *ϕ*(*X*) can be very high, sometimes even infinite. As such, a direct decomposition is not feasible computationally.

The problem can be solved by noting that the target of nonlinear DR estimation are the inner products 〈*β_j_,ϕ*(*X*)〉, rather than *β_j_* themselves. Then, given a pre-specified kernel function *k*, these inner products can be obtained by solving a dual problem: 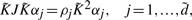 , where 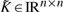 is the centered kernel matrix and *J* is a method-specific *n*×*n* matrix. Zhu and Li (2011) gave for the specification of *J* matrix for different SDR methods, including the one for kernel SIR that will be used in the numerical studies of this article. Then, for a new observation 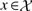 , 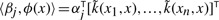 , where 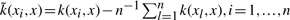 . So the inner product 〈*β_j_,ϕ*(*x*)〈 can be obtained from the kernel *k* and *α_j_*s. It is noted that the proposed nonlinear DR approach only involves decomposition of an *n*×*n* matrix, so it can handle *n < p*. Its flexible reduction form beyond the linear combination is also expected to facilitate DR. For the illustrative example considered above, if one uses a quadratic kernel, then only one linear combination in the mapped feature space is needed to summarize all regression information, and thus substantial reduction is achieved.

### 2.2 Kernel-based on Markov chain

Critical to success of any kernel-based methods for genotype data analysis is the design of kernels that can effectively capture genomic similarity (Schaid, 2010a,b). The most popular Gaussian kernel works well for continuous predictors but can perform poorly on categorical predictors such as SNPs. Some specialized kernels have been crafted for SNP data. For instance, the identity-by-state (IBS) kernel (Wessel and Schork, 2006) calculates the distance between two individuals with genotype vectors ***x****_i_* and ***x****_j_* coded by numbers of minor alleles as

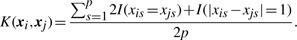

The more general weighted IBS kernel (Kwee *et al.*, 2008; Wu *et al.*, 2010) takes the form

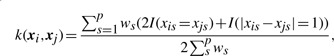

which offers flexibility of incorporating variant specific weights into kernel. Kwee *et al.* (2008) and Wu *et al.* (2010) use 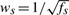 where *f_s_* is the MAF of the variant, up-weighting the importance of rare variants.

IBS and weighted IBS kernels can be regarded as sums of simple similarities evaluated at each individual SNP, which, however, may result in loss of power when complex interactions dominate the genetic effect of the SNP set. This motivates us to propose new kernels for genotype data.

We first summarize some useful devices for forming Mercer (symmetric and positive definite) kernels. Suppose *k* is a possibly asymmetric kernel and *K* is the corresponding kernel matrix with all eigenvalues positive, then

1. (*K* +*K^T^*)/2 is symmetric and positive definite;
2. *KK^T^* is a symmetric and positive definite;
3. *K* ○ *K^T^* is symmetric and positive definite, where ○ denotes the element-wise Hadamad product of two matrices;
4. If another *Q* is a kernel matrix with positive eigenvalues, then *KQ* is a kernel with positive eigenvalues.

Fact 1 follows from an inequality due to Fan (Marshall *et al.*, 2011, Theorem F.1, p. 324). Fact 2 is trivial. Fact 3 can be shown based on the Shur's inequality (Scholkopf and Smola, 2001, Proposition 13.2). Fact 4 follows from the inequality H.1.i of Marshall *et al.* (2011). These rules will be used as we construct Markov kernels below. Reader are referred to Scholkopf and Smola (2001) Chapter 13 for more basic tricks for constructing kernels.

Since SNP values are all dichotomous or trichotomous, we next focus on kernels that are built upon the discrete state space 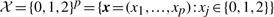 . Our new kernel for genotype data is derived from Markov chains on 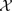 . The key idea is that the transition kernel of many Markov chains with state space 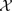 defines a Mercer kernel after appropriate transformations. This opens the door to create more informative kernels for data in a non-standard space 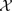 . Specifically, our new kernel is based on the well-known multi-allele Wright–Fisher (WF) process (Ewens, 2004) in population genetics. Therefore, we call this the WF kernel. Each locus is coded by the number of ‘major’ alleles and the genotype vector 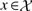 for a SNP set is modeled by a Dirichlet-Multinomial distribution with parameter ***α*** = (*α*_1_,...,*α_p_*), which can be efficiently estimated from genotypes of all individuals (Zhou and Lange, 2010). The transition kernel of WF process between two genotype vectors 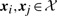 is

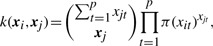

where *π* (*x_it_*) = (*x_it_* + *α_t_*)*/N* +|***α***| and |***α***| = ∑*_t=_*_1_^*p*^α*_t_*. *k* is an irreversible Markov transition kernel and its stationary distribution is not known explicitly but can be well approximated by a Dirichlet-multinomial distribution with parameter ***α***. It is well known that *k* has positive eigenvalues *λ_i_* = (2*p*)_[*i*]_/(2*p*)*^i^* = 2*p*(2*p*−1)···(2*p*−*i*+1)/(2*p*)*^i^* for *i* = 0,...,2*p*−1 (Cannings, 1974). Therefore, we aim to form a Mercer kernel by scaling and symmetrizing *k*. For large *p*, the entries of the corresponding kernel matrix *K* is small. To achieve better scaling, we normalize each column of *K* by dividing its *ℓ*_2_ norm. The column-scaled matrix is denoted by 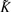 . By Fact 4 above, we know 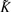 has positive eigenvalues. Then, by Facts 1 and 2, either the additive or multiplicative symmetrization gives a symmetric kernel matrix with all positive eigenvalues: 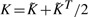 or 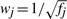 . The simulation study in Section 3.3 shows the promise of the WF kernel combined with nonlinear DR method proposed in Section 2.

### 2.3 A joint association testing strategy

Based on the nonlinear DR methods and the new kernel, we propose a joint strategy to identify SNP sets that are associated with a trait of interest in Algorithm 1. Several remarks are in order. First, as has been mentioned above, the nonlinear DR methods can be performed even if the sample size, *n*, is smaller than the number of predictors, *p*. Therefore, the entire strategy handles the situation when *n* is smaller than the number of SNPs in any candidate SNP sets. Second, the non-genetic covariates, if any, can be naturally adjusted in the GLM modeling step. Third, both the DR and the GLM modeling step permit different types of trait response. For a continuous trait, GLM reduces to a linear regression, and for a typical case–control study, it becomes a binomial model with a logit link, which leads to a LR.

## 3 RESULTS

### 3.1 Data description

**Fig. 1.**
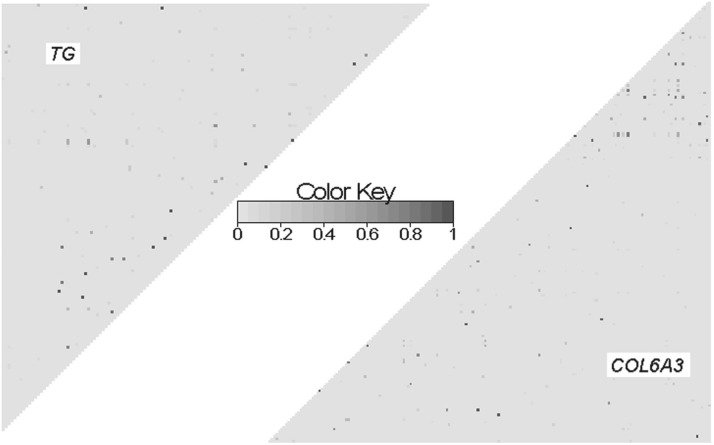
LD structures of the 146 SNPs in *TG* (top left) and 187 SNPs in *COL6A3* (bottom right)

**Fig. 2.**
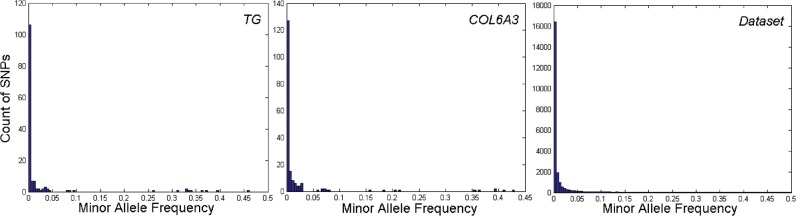
Histograms of MAFs for SNPs in *TG* (left), *COL6A3* (middle) and the entire GAW17 dataset (right)


We have performed simulation studies to illustrate the promise of the information aggregation method using the nonlinear DR and the WF kernel discussed above. These studies use real genotype data of 697 individuals compiled from the 1000 Genome Project (2010) by the Genetic Analysis Workshop 17 (GAW17). We investigate the empirical power and Type-I error of our method on the basis of the LD structure of two genes, *TG* and *COL6A3*. The two genes are used because mutations in these genes have been found to cause certain diseases. *TG* encodes a protein called thyroglobulin; mutations in this gene have been found related to congenital hypothyroidism and autoimmune disorders (http://ghr.nlm.nih.gov/gene/TG). *COL6A3* encodes one component of Type-VI collagen; mutations in this gene have been found related to Bethlem myopathy and Ullrich congenital muscular dystrophy (http://ghr.nlm.nih.gov/gene/COL6A3). In the dataset, *TG* contains 146 SNPs, among which 10 are common variants (MAF*>*10%) and 113 are rare variants (MAF*<*1%); *COL6A3* contains 187 SNPs, among which 10 are common variants and 143 are rare variants. In the two genes, only a few pairs of SNPs have high LD (Fig. 1). Figure 2 provides a comparison of the MAF distribution of *TG* and *COL6A3* with that of the entire dataset. The three distributions agree well except that *TG* and *COL6A3* have slightly higher percentage of common variants.

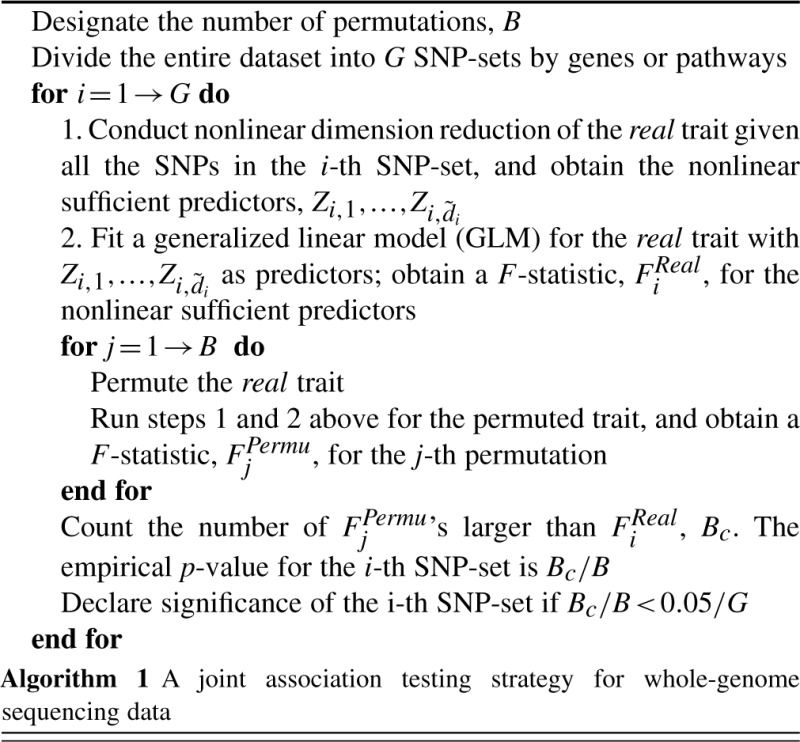


### 3.2 Simulation setup

In order to make a comprehensive performance comparison between the proposed methods and existing ones, a variety of true genetic effects are examined in different simulation studies (Table 1). For each study, a total of 1000 replicates are simulated. In each replicate, a quantitative trait is first simulated under the null model *Q*_0_ = *∈*, where *∈* is a standard normal noise. The top 50% of the distribution of *Q*_0_ are then declared affected, by which we define a binary disease status. It is easy to see that, under the null model, the quantitative trait and disease status do not depend on genotypes.

**Table 1.** Results of simulation studies based on sequence data of genes *TG* and *COL*6A3

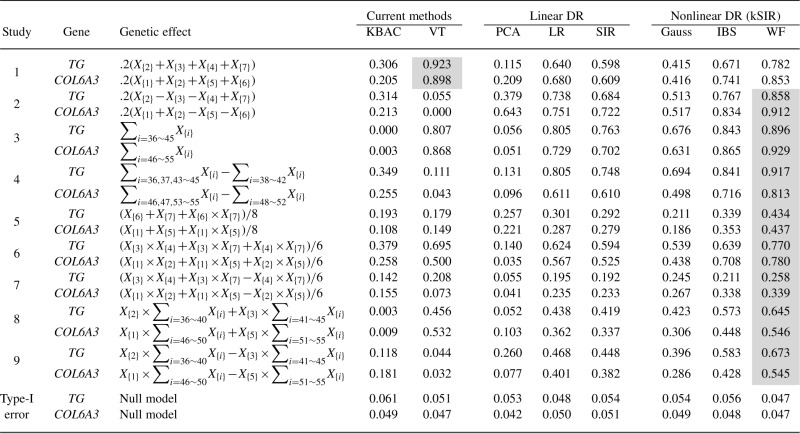

Each study focuses on one genetic model mimicking a specific type of true genetic effect. Under ‘Genetic Effect’ are the true genetic effects that generate the quantitative trait, where *X*_{j}_'s are SNPs in descending order according to their MAFs. The common variants (*j* ≤ 10) are selected to have low pairwise LD. The binary trait is determined from the quantitative trait and serves as the response variable in the simulation studies. The numbers under the names of different methods are their empirical power in different studies or Type-I error.

Then, under the alternative model, a quantitative trait is generated according to
(3)
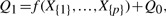

where *X*_{*j*}_'s are SNPs in descending order according to their MAFs and *f* is the true genetic effect model, which differs among simulation scenarios. A binary disease status based on *Q*_1_ is defined in a similar way as *Q*_0_. For reference purpose, if in Equation (3) *f* = ∑_*j=*__1_*^p^ β_j_X*_{*j*}_ is a linear additive model, then conditional on the fact that this is an half-affected-half-control sample and assuming that existence of causal SNPs in the gene does not heavily influence the general cutoff to *Q*_1_ (which is the case for most of our simulation studies shown below), there is an approximate correspondence between coefficient *β_j_* and odds ratio of the SNP *X*_{*j*}_: odds ratio ≈ (1−*Φ*(−*β_j_*;0,1))*/Φ*(−*β_j_*;0,1), where *Φ*(*x*;*μ,σ*) represents cumulative normal distribution function with mean *μ* and standard deviation *σ*. Therefore, a coefficient *β* from 0.2 to 1 corresponds to an odds ratio from 1.4 to 5.3. On the contrary, a coefficient *β* from −0.2 to −1 reduces odds ratio to 0.7–0.2.

### 3.3 Simulation results

We compared our method of DR followed by LR model (a special case of GLM for binary trait) with kernel-based adaptive cluster (KBAC) method of Liu and Leal (2010) and variable threshold (VT) test of Price *et al.* (2010). For KBAC, if a SNP set contains only rare variants, we adopted a permutation test for the KBAC statistic; otherwise, following Liu and Leal (2010), a LR model was used to incorporate both the common variants and a variable for the kernel weight generated by KBAC. Standard permutation procedure was then applied to evaluate the significance. VT test can be used regardless of the existence of common variants. We also compared some variants at the step of DR, namely PCA, LR for testing all SNPs, SIR and kernel SIR (kSIR) with various kernel functions (Gaussian, IBS and WF). Multiplicative symmetrization was performed for WF kernel. For each of the approaches under comparison, 10 000 permutations were used to generate a null distribution for the test statistic. For each DR-based method, the leading summary variable was used to represent the SNP set. For each method under comparison, an empirical power was then obtained by counting how many of empirical *P*-values over 1000 replications are less than a nominal significance level of *α* = 0.05 under the alternative model using the designated causal SNPs. An empirical Type-I error was evaluated in a similar fashion except that the trait was generated under the null model. As mentioned above, we designed various scenarios to evaluate methods. Results are summarized in Table 1.

#### 3.3.1 Empirical Type-I error

The last two rows of Table 1 show that the empirical Type-I errors of all the methods are relatively close to the nominal significance level of 0.05.

#### 3.3.2 Empirical power comparison

Simulations 1–4 represent main effect models. In Simulations 1 and 2, informative SNPs are all common variants, whose MAFs all fall into the range of 0.32–0.43. When all SNPs are deleterious (Simulation 1), VT outperforms KBAC, linear and nonlinear DR methods. However, when two of the informative alleles are protective (Simulation 2), the performance of VT drops dramatically, while the performance of the other methods does not. In this case, WF works the best and IBS is the second. In Simulation 3 and 4, informative SNPs are all rare variants, whose MAFs are between 0.005 and 0.0086. Note that few people have totally more than one minor alleles over these 10 SNPs. No matter there are protective rare variants or not, WF and IBS have the best and second best performance, respectively. VT has a comparable performance when there are only deleterious effect (Simulation 3), but the existence of protective variants destroys its performance again (Simulation 4). Note that under these linear genetic models, the WF and IBS kSIR's still work better than the LR and linear SIR, while the Gaussian kSIR does not. This reflects the superiority of these kernels for sequence data.

Simulation 5 represents a model with both main and epistasis effects. For each gene, both of the two informative SNPs are common variants with very low LD. In this case, WF works better than IBS, which then outperforms KBAC, VT and other DR-based methods. Simulations 6 and 7 are pure epistasis models among three common SNPs with low LD between each other, while Simulations 8 and 9 represent epistasis between common and rare variants. It is clear that nonlinear DR methods with WF or IBS kernel outperform linear dimension reduction methods as well as KBAC and VT. KBAC loses most of its power in detecting common and rare variant interactions. VT performs poorly when there are protective effects.

Weighted IBS kernel using weights 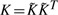 is also evaluated as a candidate kernel for kSIR. The performance of weighted IBS, however, is found worse than that of IBS for most of the studies in Table 1 (results not shown).

**Fig. 3.**
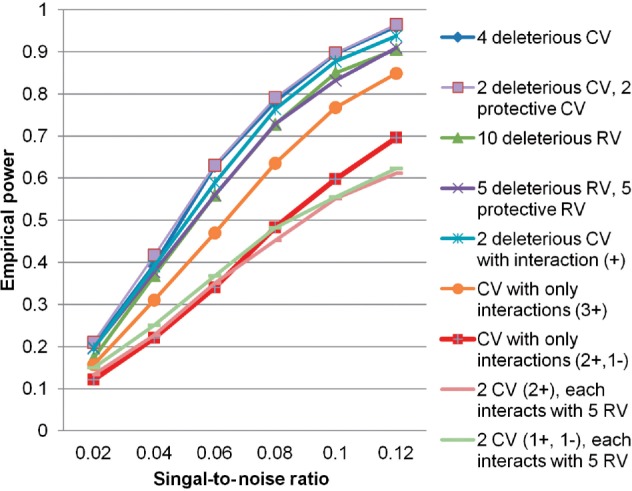
Empirical power of kSIR with WF kernel varies with SNR. Gene *TG* is used for simulation. The nine scenarios in Table 1 are examined. CV, common variant; RV, rare variant

We further evaluated the performance of kSIR with WF kernel at different signal-to-noise ratios (SNRs) using gene *TG*. For Model (3), SNR is defined as the ratio of the variance of the signal *f* and the error variance. A number of SNR values were simulated by multiplying *f* with different coefficients. Figure 3 shows the empirical power for the nine scenarios that are given in Table 1 with the SNRs ranging from 0.02 to 0.12. It is seen that the empirical power exceeds 0.8 for additive effects with 0.1 SNR, including the ones composed of protective and/or rare variants. The complex interaction effects are less detectable than simple additive effects. Nevertheless, when the SNR reaches 0.12, the empirical power exceeds 0.7 and 0.6 for common–common and rare–common interactions, respectively.

In conclusion, VT is extremely vulnerable to protective variants and less sensitive to epistasis effects. KBAC does not capture interactions between rare and common variants. Even if the true genetic model is additive, linear DR-based methods do not outperform nonlinear DR methods with kernels specifically designed for genotype data. Among the different kernel methods, the proposed WF kernel always outperforms IBS kernel, which then works better than Gaussian kernel.

## 4 DISCUSSION

Ideally, an association test should be able to handle: (i) high dimensionality of genomic dataset, which typically far exceeds the sample size; (ii) both rare and common variants; (iii) additive, recessive and dominant models of gene action; (iv) both quantitative traits and disease dichotomies and (v) non-genetic covariates. Most of existing solutions could not deliver across the board judged by all those criteria. To bridge the gap, we have proposed a strategy based on nonlinear supervised DR and a new kernel to effectively aggregate genotype information. Such an aggregation increases the likelihood of detecting multiple causal variants, whereas nonlinear reduction permits complex interactive relationship among genetic variants. Moreover, the GLM framework based on the aggregated features can naturally handle both quantitative and categorical traits, and incorporate non-genetic predictors and/or environmental variables. Finally, the proposed WF kernel based on Markov chain theory for genotype data has been proven useful compared with some existing kernels and can potentially benefit a wide range of kernel methods, such as kernel PCA, support vector machines and nonparametric and semiparametric regressions.

There are a number of possible avenues for future extensions. First, a number of Markov chain kernels in addition to the WF kernel can be explored, for instance, Ehrenfest kernels, hypergeometric kernels and Dirichlet-Multinomial kernels (Khare and Zhou, 2009; Zhou and Lange, 2009). Second, our current aggregation strategy has not taken advantage of any available information regarding the relationships among SNPs within a SNP set, e.g. their genetic distances. Incorporating those information can potentially facilitate the design of more effective kernels.
